# Supplementary material for: Natural variation in teosinte at the domestication locus teosinte branched1 (tb1)
Source: PeerJ. 2015 Apr 16;3:e900. doi: 10.7717/peerj.900 (PMC4406365; doi:10.7717/peerj.900)
Supplement: Table S1 — Accessions of Zea mays ssp. mexicana (RIMME) and Zea mays ssp. parviglumis (RIMPA) sampled. RIHY is a Z. mays ssp. parviglumis and Zea mays ssp. mays hybrid. [file peerj-03-900-s001.pdf]

Table S1: Accessions of *Zea mays* ssp. *mexicana* (RIMME) and *Zea mays* ssp. *parviglumis* (RIMPA) sampled. RIHY is a *Z. mays* ssp. *parviglumis* and *Zea mays* ssp. *mays* hybrid.

Accession	USDA ID	Population	Alleles Sampled	*Hopscotch* Freq.	No *Hopscotch* Freq.
RIHY0009	N/A	N/A	2	0.5	0.5
RIMME0006	PI 566673	Durango	2	0	1
RIMME0007	PI 566680	Guanajuato	2	0	1
RIMME0008	PI 566681	Michoacan	2	0	1
RIMME0009	PI 566682	Distrito Federal	2	0	1
RIMME0011	PI 566685	Mexico	2	0	1
RIMME0014	Ames 28398	TIL25 Jalisco	6	0	1
RIMME0017	Ames 699874	Ayotlan	8	0	1
RIMME0021	N/A	El Porvenir	69	0.17	0.83
RIMME0026	N/A	Opopeo	42	0.07	0.93
RIMME0028	N/A	Puruandiro	28	0.04	0.96
RIMME0029	N/A	Ixtlan	35	0	1
RIMME0030	N/A	San Pedro	27	0	1
RIMME0031	N/A	Tenango del Aire	25	0.08	0.92
RIMME0032	N/A	Nabogame	24	0	1
RIMME0033	N/A	Puerta Encantada	25	0	1
RIMME0034	N/A	Santa Clara	23	0	1
RIMME0035	N/A	Xochimilco	25	0	1
RIMPA0001	Ames 21786	El Salado	4	0	1
RIMPA0003	Ames 21789	Mazatlan	8	0.13	0.87
RIMPA0017	Ames 21814	N/A	4	0	1
RIMPA0019	Ames 21826	El Salado	2	0.50	0.50
RIMPA0029	Ames 21853	N/A	2	0.50	0.50
RIMPA0031	Ames 21856	N/A	2	0.5	0.5
RIMPA0035	Ames 21889	Jalisco	4	0	1
RIMPA0040	PI 384063	Mexico	4	0	1
RIMPA0042	PI 384065	Guerrero	4	0.25	0.75
RIMPA0043	PI 384066	Guerrero	4	0	1
RIMPA0045	PI 384071	Guerrero	4	0	1
RIMPA0055	Ames 28399	TIL01, Michoacan	2	0	1
RIMPA0056	Ames 28400	TIL03 Jalisco	2	0.50	0.50
RIMPA0057	Ames 28401	TIL06 Guerrero	2	0.50	0.50
RIMPA0058	N/A	N/A	4	0.50	0.50
RIMPA0059	N/A	N/A	4	1	0
RIMPA0060	Ames 28404	TIL10 Guerrero	2	0	1
RIMPA0061	Ames 28405	TIL11 Nayarit	4	0.5	0.5
RIMPA0062	Ames 28406	TIL14 Jalisco	4	0.5	0.5
RIMPA0063	Ames 28407	TIL15 Guerrero	4	0	1
RIMPA0064	Ames 28408	TIL16 Guerrero	3	0	1
RIMPA0065	Ames 28409	TIL17 Guerrero	4	0.25	0.75
RIMPA0068	Ames 28066	Jalisco, Mexico	16	0	1
RIMPA0069	Ames 28067	Ixtlan	14	0.14	0.86
RIMPA0070	Ames 28068	Benito Jaurez	16	0	1
RIMPA0071	Ames 28069	Tuzantla	28	0	1
RIMPA0072	Ames 28070	Tiquicheo	16	0	1
RIMPA0073	Ames 28071	Tiquicheo	16	0.12	0.88
RIMPA0074	Ames 28072	Huetamo	12	0	1
RIMPA0075	Ames 28073	Huetamo	2	0	1
RIMPA0076	Ames 28074	Huetamo	4	0	1
RIMPA0077	Ames 28075	Caracuaro	2	0	1
RIMPA0078	Ames 28076	Caracuaro	2	0.5	0.5
RIMPA0079	Ames 28077	Villa Madero	14	0	1
RIMPA0080	Ames 28078	Guachinango	12	0	1
RIMPA0081	Ames 28080	Ameca	16	0	1
RIMPA0083	Ames 28082	Tepoztlan	14	0	1
RIMPA0084	Ames 28083	Tepoztlan	16	0	1
RIMPA0085	Ames 28084	Miahuatlan	16	0	1
RIMPA0086	Ames 28085	Miahuatlan	16	0.06	0.94
RIMPA0087	Ames 28086	Tecoanapa	24	0	1
RIMPA0089	Ames 28088	Guerrero	12	0	1
RIMPA0090	Ames 28089	Guerrero	10	0	1
RIMPA0091	Ames 28090	Guerrero	16	0	1
RIMPA0092	Ames 28091	Guerrero	10	0	1

Accession	USDA ID	Population	Alleles Sampled	*Hopscotch* Freq.	No *Hopscotch* Freq.
RIMPA0093	Ames 28092	Guerrero	26	0.08	0.92
RIMPA0094	Ames 28093	Guerrero	2	0	1
RIMPA0095	Ames 28094	Guerrero	4	0	1
RIMPA0096	Ames 28095	Guerrero	26	0.04	0.96
RIMPA0097	Ames 28096	Guerrero	6	0	1
RIMPA0098	Ames 28097	Guerrero	4	0	1
RIMPA0099	Ames 28098	Guerrero	4	0	1
RIMPA0100	Ames 28099	Guerrero	6	0	1
RIMPA0101	Ames 28100	Guerrero	2	0	1
RIMPA0103	Ames 28102	Guerrero	2	0	1
RIMPA0104	Ames 28103	Guerrero	22	0.09	0.91
RIMPA0105	Ames 28104	Guerrero	6	0	1
RIMPA0106	Ames 28105	Guerrero	6	0.33	0.67
RIMPA0107	Ames 28106	Guerrero	4	0	1
RIMPA0108	Ames 28107	Guerrero	6	0	1
RIMPA0109	Ames 28108	Michoacan	4	0.25	0.75
RIMPA0110	Ames 28109	Michoacan	2	0	1
RIMPA0111	Ames 28110	Michoacan	4	0	1
RIMPA0112	Ames 28111	Michoacan	4	0.25	0.75
RIMPA0114	Ames 28113	Michoacan	6	0.17	0.83
RIMPA0116	Ames 28115	Mexico	2	0	1
RIMPA0117	Ames 28116	Mexico	4	0	1
RIMPA0118	Ames 28117	Mexico	6	0.17	0.83
RIMPA0119	Ames 28118	Mexico	2	0	1
RIMPA0120	Ames 28119	Mexico	1	1	0
RIMPA0121	Ames 28120	Mexico	2	0	1
RIMPA0128	Ames 28127	Mexico	2	0.5	0.5
RIMPA0129	Ames 28128	Michoacan	2	0.5	0.5
RIMPA0135	Ames 28134	Nayarit	24	0	1
RIMPA0138	Ames 28137	Jalisco	2	0.5	0.5
RIMPA0139	Ames 28138	Jalisco	1	1	0
RIMPA0142	Ames 28141	Colima	18	0.44	0.56
RIMPA0144	Ames 28143	Jalisco	2	1	0
RIMPA0145	Ames 28144	Michoacan	1	1	0
RIMPA0147	Ames 28146	Jalisco	1	1	0
RIMPA0155	N/A	Jalisco	73	0.01	0.99
RIMPA0156	N/A	Jalisco	20	0	1
RIMPA0157	N/A	Jalisco	58	0.34	0.66
RIMPA0158	N/A	Jalisco	64	0.53	0.47
RIMPA0159	N/A	Jalisco	26	0	1
RIMPA0162	Ames 21785	N/A	4	0	1
