# Supplementary material for: Natural variation in teosinte at the domestication locus teosinte branched1 (tb1)
Source: PeerJ. 2015 Apr 16;3:e900. doi: 10.7717/peerj.900 (PMC4406365; doi:10.7717/peerj.900)
Supplement: Table S2 — Hopscotch frequency in sampled Zea mays ssp. mays (RIMMA). [file peerj-03-900-s002.pdf]

Table S2: *Hopscotch* frequency in sampled *Zea mays* ssp. *mays* (RIMMA).

Accession	USDA ID	Alleles Sampled	*Hopscotch* Freq.
RIMMA0066	Ames 4327	2	1
RIMMA0075	Ames 19309	2	1
RIMMA0077	Ames 19313	2	1
RIMMA0079	Ames 19321	2	1
RIMMA0081	Ames 20190	2	1
RIMMA0084	Ames 22439	2	1
RIMMA0086	Ames 22748	2	1
RIMMA0088	Ames 22756	2	1
RIMMA0089	Ames 23392	2	1
RIMMA0090	Ames 23423	2	1
RIMMA0092	Ames 23427	4	1
RIMMA0094	Ames 23447	4	1
RIMMA0097	Ames 23488	2	1
RIMMA0099	Ames 23509	2	1
RIMMA0100	Ames 23518	2	1
RIMMA0101	Ames 23519	2	1
RIMMA0104	Ames 24590	2	1
RIMMA0108	Ames 26022	2	1
RIMMA0111	Ames 26770	6	1
RIMMA0115	Ames 26798	2	1
RIMMA0117	Ames 27045	2	1
RIMMA0130	Ames 28336	2	1
RIMMA0133	NSL 8581	2	1
RIMMA0134	NSL 22632	2	1
RIMMA0135	NSL 22634	2	1
RIMMA0142	NSL 30060	2	0.5
RIMMA0143	NSL 30065	4	1
RIMMA0146	NSL 30863	4	1
RIMMA0149	NSL 30869	2	1
RIMMA0152	NSL 32722	2	1
RIMMA0153	NSL 32728	2	1
RIMMA0154	NSL 32732	2	1
RIMMA0155	NSL 42809	2	1
RIMMA0156	NSL 42872	2	1
RIMMA0157	NSL 42874	2	1
RIMMA0158	NSL 42875	2	1
RIMMA0159	NSL 42878	2	1
RIMMA0160	NSL 65864	2	1
RIMMA0162	NSL 65866	2	1
RIMMA0166	PI 213696	2	1
RIMMA0167	PI 213697	2	1
RIMMA0168	PI 213698	2	1
RIMMA0169	PI 213699	2	1
RIMMA0172	PI 213705	2	1
RIMMA0174	PI 213708	4	1
RIMMA0177	PI 213715	2	1
RIMMA0178	PI 213716	2	1
RIMMA0179	PI 213717	2	1
RIMMA0181	PI 213719	2	1
RIMMA0183	PI 213721	2	1
RIMMA0184	PI 213722	2	1
RIMMA0186	PI 213724	2	1
RIMMA0187	PI 213725	1	1
RIMMA0188	PI 213727	2	1
RIMMA0195	PI 213781	2	1
RIMMA0196	PI 213793	2	1
RIMMA0197	PI 214197	2	1
RIMMA0198	PI 214293	2	1
RIMMA0199	PI 214294	2	1
RIMMA0200	PI 214295	2	1
RIMMA0202	PI 217407	2	1
RIMMA0203	PI 217408	2	1
RIMMA0206	PI 217473	2	1
RIMMA0208	PI 218005	2	1
RIMMA0209	PI 218195	2	1

Accession	USDA ID	Alleles Sampled	*Hopscotch* Freq.
RIMMA0210	PI 219871	2	1
RIMMA0212	PI 219882	2	1
RIMMA0213	PI 219883	2	1
RIMMA0214	PI 219884	2	1
RIMMA0217	PI 221872	2	1
RIMMA0218	PI 221873	2	1
RIMMA0220	PI 221876	2	1
RIMMA0221	PI 221877	2	1
RIMMA0222	PI 221878	2	1
RIMMA0223	PI 221880	2	1
RIMMA0226	PI 221889	2	1
RIMMA0227	PI 222314	2	1
RIMMA0228	PI 222315	2	1
RIMMA0229	PI 222316	2	1
RIMMA0230	PI 222317	2	1
RIMMA0232	PI 222469	2	1
RIMMA0233	PI 222470	2	1
RIMMA0235	PI 222474	2	0.5
RIMMA0242	PI 222614	2	1
RIMMA0243	PI 222615	2	1
RIMMA0247	PI 222639	4	1
RIMMA0248	PI 222640	2	1
RIMMA0249	PI 222641	2	1
RIMMA0252	PI 233002	2	1
RIMMA0253	PI 233006	2	1
RIMMA0254	PI 233008	2	1
RIMMA0256	PI 237000	2	1
RIMMA0257	PI 257506	2	1
RIMMA0258	PI 267179	2	1
RIMMA0259	PI 269743	2	1
RIMMA0260	PI 269744	2	1
RIMMA0262	PI 270297	2	1
RIMMA0263	PI 278713	2	1
RIMMA0264	PI 278716	2	1
RIMMA0265	PI 278717	2	1
RIMMA0268	PI 278721	2	1
RIMMA0269	PI 278724	2	1
RIMMA0270	PI 280061	2	1
RIMMA0272	PI 280853	2	1
RIMMA0275	PI 311232	2	1
RIMMA0276	PI 311235	2	1
RIMMA0277	PI 311236	2	1
RIMMA0279	PI 311241	2	1
RIMMA0280	PI 311243	2	1
RIMMA0283	PI 401757	2	1
RIMMA0285	PI 414176	2	1
RIMMA0288	PI 452032	2	1
RIMMA0290	PI 452045	2	1
RIMMA0291	PI 452046	2	1
RIMMA0292	PI 452047	2	1
RIMMA0293	PI 452048	2	1
RIMMA0298	PI 483273	2	1
RIMMA0302	PI 540784	2	1
RIMMA0305	PI 548792	2	1
RIMMA0310	PI 550462	2	1
RIMMA0312	PI 550466	2	1
RIMMA0320	PI 583896	2	1
RIMMA0322	PI 583898	2	1
RIMMA0324	PI 587125	2	1
RIMMA0334	PI 587153	2	1
RIMMA0336	PI 607519	2	1
RIMMA0337	PI 607522	2	1
RIMMA0338	PI 607593	2	1
RIMMA0339	PI 607599	2	1
RIMMA0340	PI 607602	2	1
RIMMA0341	PI 608465	2	1
RIMMA0342	PI 608502	2	1

Accession	USDA ID	Alleles Sampled	*Hopscotch* Freq.
RIMMA0344	PI 608539	2	1
RIMMA0346	PI 608618	2	1
RIMMA0347	PI 608619	2	1
RIMMA0350	PI 608646	2	1
RIMMA0351	PI 608652	2	1
RIMMA0352	PI 608764	2	1
RIMMA0357	PI 608777	2	1
RIMMA0360	N/A	18	1
RIMMA0362	N/A	8	1
RIMMA0363	N/A	16	1
RIMMA0370	N/A	17	1
RIMMA0372	N/A	24	1
RIMMA0373	N/A	22	1
RIMMA0408	PI 478967	2	1
RIMMA0411	PI 479044	1	1
RIMMA0413	PI 484413	1	1
RIMMA0414	PI 484421	1	1
RIMMA0424	PI 485120	8	0.9
RIMMA0432	PI 489126	1	1
RIMMA0434	PI 490921	2	0.5
RIMMA0435	PI 490973	1	1
RIMMA0442	PI 537097	2	1
RIMMA0443	PI 537099	2	1
RIMMA0444	PI 538009	2	1
RIMMA0445	PI 538011	2	1
RIMMA0446	PI 539920	2	1
RIMMA0447	PI 539921	2	1
RIMMA0448	PI 539922	2	1
RIMMA0449	PI 539923	2	1
RIMMA0450	PI 539924	2	1
RIMMA0451	PI 539926	2	1
RIMMA0452	PI 539927	2	1
RIMMA0453	PI 543843	2	1
RIMMA0454	PI 543846	2	1
RIMMA0455	PI 543847	2	1
RIMMA0456	PI 543849	2	1
RIMMA0457	PI 543850	2	1
RIMMA0458	PI 547086	2	1
RIMMA0459	PI 547088	2	1
RIMMA0483	PI 600954	2	1
RIMMA0490	PI 601003	2	1
RIMMA0515	PI 601270	2	1
RIMMA0537	PI 601416	2	1
RIMMA0550	PI 601468	2	1
RIMMA0553	PI 601489	2	1
RIMMA0559	PI 601495	2	1
RIMMA0561	PI 601497	2	1
RIMMA0562	PI 601498	2	1
RIMMA0571	PI 601562	2	1
RIMMA0572	PI 601563	2	1
RIMMA0577	PI 601568	2	1
RIMMA0579	PI 601570	2	1
RIMMA0590	PI 601724	2	1
RIMMA0591	PI 601725	2	1
RIMMA0592	PI 601729	2	1
RIMMA0593	PI 601773	2	1
RIMMA0594	PI 601774	2	1
RIMMA0595	PI 601775	2	1
RIMMA0596	PI 601776	2	1
RIMMA0597	PI 601777	2	1
RIMMA0598	PI 601778	2	1
RIMMA0599	PI 601779	2	1
RIMMA0600	PI 601780	2	1
RIMMA0601	PI 601781	2	1
RIMMA0602	PI 601782	2	1
RIMMA0603	PI 601783	2	1
RIMMA0604	PI 601784	2	1

Accession	USDA ID	Alleles Sampled	*Hopscotch* Freq.
RIMMA0605	PI 601785	2	1
RIMMA0606	PI 601786	2	1
RIMMA0607	PI 601787	2	1
RIMMA0608	PI 601788	2	1
RIMMA0609	PI 601789	2	1
RIMMA0610	PI 601790	2	1
RIMMA0611	PI 601791	2	1
RIMMA0612	PI 601792	2	1
RIMMA0613	PI 601808	2	1
RIMMA0622	PI 645837	2	0.5
RIMMA0624	PI 645930	1	1
RIMMA0629	PI 646057	2	0.5
RIMMA0631	PI 646072	2	0.5
RIMMA0659	NSL 286729	2	1
RIMMA0660	PI 485451	2	1
RIMMA0678	NSL 285833	1	1
RIMMA0681	PI 628446	2	1
RIMMA0683	PI 483568	2	1
RIMMA0684	Ames 28482	2	1
RIMMA0685	NSL 287623	4	1
RIMMA0693	PI 487959	2	1
RIMMA0694	Ames 28487	2	1
RIMMA0695	Ames 28567	1	1
RIMMA0699	Ames 28465	2	1
RIMMA0704	Ames 28623	2	1
RIMMA0706	Ames 28615	2	1
RIMMA0711	Ames 29075	1	1
RIMMA0713	PI 489399	2	1
RIMMA0715	Ames 28454	2	1
RIMMA0717	PI 483926	2	1
RIMMA0718	PI 484805	2	1
RIMMA0723	Ames 28565	2	1
RIMMA0724	PI 490826	2	1
RIMMA0725	PI 490827	1	1
RIMMA0728	PI 484969	1	1
RIMMA0732	Ames 28456	2	1
RIMMA0734	Ames 26028	2	1
RIMMA0738	N/A	2	1
RIMMA0739	N/A	2	1
RIMMA0744	N/A	2	1
RIMMA0747	N/A	1	1
RIMMA0748	N/A	2	1
RIMMA0749	N/A	2	1
RIMMA0750	N/A	2	0.5
RIMMA0751	N/A	1	1
RIMMA0752	N/A	1	1
RIMMA0753	N/A	2	1
RIMMA0755	N/A	2	1
