# Supplementary figures and images for: Natural variation in teosinte at the domestication locus teosinte branched1 (tb1)
Source: PeerJ. 2015 Apr 16;3:e900. doi: 10.7717/peerj.900 (PMC4406365; doi:10.7717/peerj.900)

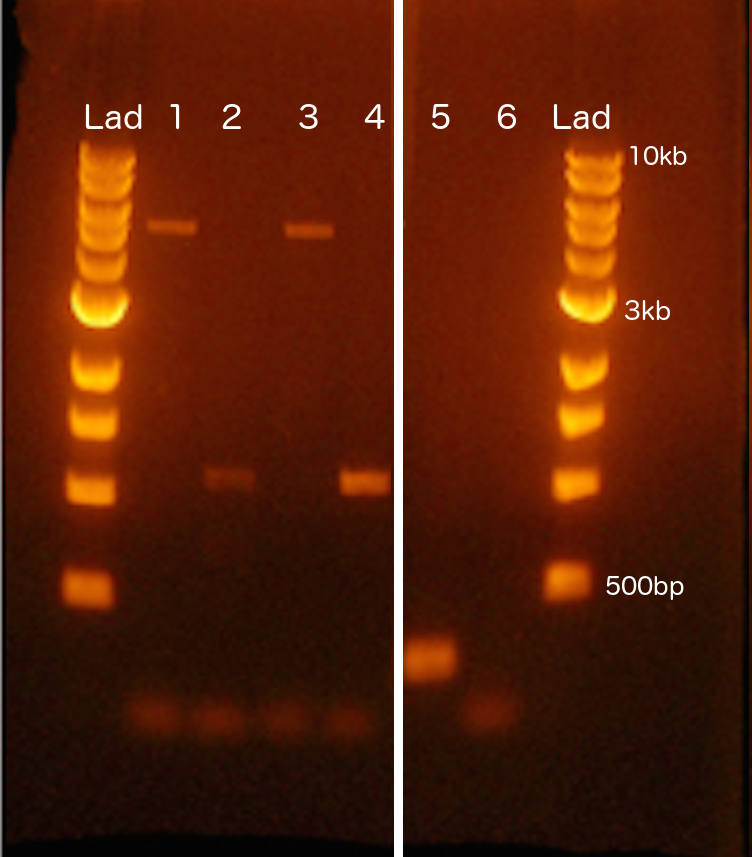

Supplement: Figure S1 — Agarose gel image of amplification products for genotyping of the Hopscotch element. Lanes 1 (HopF/HopR; 5 kb band) and 2 (HopF/HopIntR; 1.1 kb) are the products for one individual that is homozygous for the element; Lanes 3 (HopF/HopR; 5 kb band) and 4 (HopF/HopIntR; 1.1 kb) are also the products of an individual that is homozygous for the element; and Lanes 5 (HopF/HopR; 300 bp) and 6 (HopF/HopIntR; N/A) are the products of an individual that is homozygous for the teosinte (lacking the Hopscotch) allele. [file peerj-03-900-s003.png]

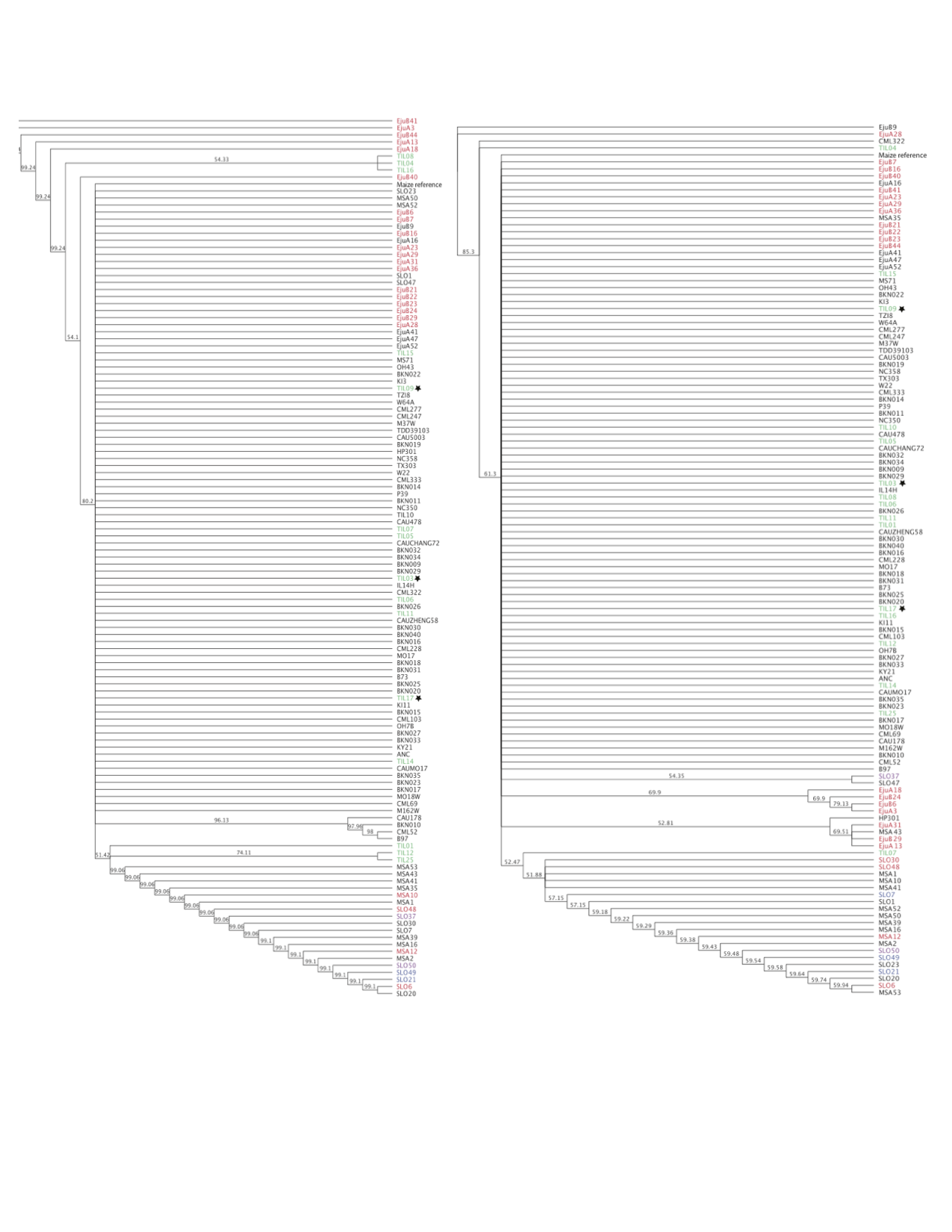

Supplement: Figure S2 — Neighbor-joining tree of the sequenced region in the 5′ UTR (right; Region 1) and the 66,169 bp upstream region (left; Region 2) of tb1 using 10,000 bootstraps. Individuals with genotype data are colored: Homozygous for the teosinte (no Hopscotch) allele (red), homozygous for the maize (Hopscotch) allele (blue), heterozygotes (purple). TILs (teosinte inbred lines) are colored in green, with stars indicating the 3 TILs known to have the Hopscotch insertion. Black indicates individuals not genotyped for the Hopscotch insertion. EjuA refers to individuals from population Ejutla A, EjuB from Ejutla B, SLO from San Lorenzo, and MSA from La Mesa. Remaining individuals are lines of maize (Zea mays ssp. mays). [file peerj-03-900-s004.png]
